# Effects of Depressive Symptoms, Feelings, and Interoception on Reward-Based Decision-Making: Investigation Using Reinforcement Learning Model

**DOI:** 10.3390/brainsci10080508

**Published:** 2020-08-01

**Authors:** Hiroyoshi Ogishima, Shunta Maeda, Yuki Tanaka, Hironori Shimada

**Affiliations:** 1 Graduate School of Human Sciences, Waseda University, 359-1192 Saitama, Japan; 2 Graduate School of Education, Tohoku University, 980-8576 Miyagi, Japan; shunta.maeda.d2@tohoku.ac.jp; 3 Faculty of Humanities, Wayo Women’s University, 272-8533 Chiba, Japan; yuk-tanaka@wayo.ac.jp; 4 Faculty of Human Sciences, Waseda University, 359-1192 Saitama, Japan; simac@waseda.jp

**Keywords:** depression, interoception, feeling, reward-based decision-making, reinforcement learning modeling

## Abstract

Background: In this study, we examined the relationships between reward-based decision-making in terms of learning rate, memory rate, exploration rate, and depression-related subjective emotional experience, in terms of interoception and feelings, to understand how reward-based decision-making is impaired in depression. Methods: In all, 52 university students were randomly assigned to an experimental group and a control group. To manipulate interoception, the participants in the experimental group were instructed to tune their internal somatic sense to the skin-conductance-response waveform presented on a display. The participants in the control group were only instructed to stay relaxed. Before and after the manipulation, the participants completed a probabilistic reversal-learning task to assess reward-based decision-making using reinforcement learning modeling. Similarly, participants completed a probe-detection task, a heartbeat-detection task, and self-rated scales. Results: The experimental manipulation of interoception was not successful. In the baseline testing, reinforcement learning modeling indicated a marginally-significant correlation between the exploration rate and depressive symptoms. However, the exploration rate was significantly associated with lower interoceptive attention and higher depressive feeling. Conclusions: The findings suggest that situational characteristics may be closely involved in reward exploration and highlight the clinically-meaningful possibility that intervention for affective processes may impact reward-based decision-making in those with depression.

## 1. Introduction

Depression has been associated with hyposensitivity to reward (for a review, see [1]). This hyposensitivity to reward might also affect reward-based decision-making in individuals with depression. Evidence suggests that depression is associated with a reduced ability to maximize rewards by decision-making (e.g., [2,3]). 

Recent research has sought an understanding of the complexity of reward-value learning and motivation using a reinforcement learning perspective [4], which refers to a psychological learning model of so-called operant learning. In such a model, it is assumed that individuals learn behavior through interacting with the environment, with a goal to maximize the reward obtained from the environment [5]. The aim of learning in this model is to minimize the experienced difference between the reward and its prediction (prediction error; [6]). Assuming that the behavior is rooted in reinforcement learning, it is possible to infer parameters for reward-value learning, such as the learning rate (or the extent of reward-value learning), which reflects the impact of reward in the last trial on the participant’s subsequent choice, and the memory rate (or the extent of adherence to past experiences), which reflects the effect of the prior reinforcement history on the choice, as well as parameters related to the individual psychological motivations that control reward learning, such as the exploration rate (or the high greediness for acquiring reward) which is expressed as low behavioral randomness and reflects whether choice was random or determined by feedback, from the behavioral pattern of a subject [7]. In other words, investigating the characteristics of reward-based decision-making in depression from the perspective of reinforcement learning may lead to a further description of the complex interactions between learning of reward value and motivation for reward acquisition.

Although multiple studies have applied reinforcement learning modeling to reward-based decision-making tasks in depression [8], the findings have not been consistent. Several studies report that depression is associated with lower levels of exploration rate in the reversal learning task, in which participants are initially trained to learn that two stimuli reliably predict two distinct outcomes, and then the two stimulus–outcome contingencies are reversed. For example, Kunisato et al. [9] showed that after the stimulus–reward contingency was reversed, the exploration rate was lower in participants with depressive symptoms than in non-depressed participants. Additionally, Huys et al. [10] conducted a Bayesian meta-analysis and observed a reduced exploration rate in major depressive disorder, although the behavioral data included in the meta-analysis was not derived from a reversal-learning task. However, the findings for the other parameters are not always consistent. For example, as for the memory rate, Dombrovski et al. [11] found that, regarding patients with depression and a history of attempted suicide, the only difference from healthy individuals was a lower memory rate after stimulus reversal. In addition, Rupprechter et al. [3] found that in simple reward-value estimations, unmedicated depression patients exhibit lower memory rates than learning rates. However, Chase et al. [12] reported no change in the learning rate after stimulus reversal in reversal-learning tasks. Similarly, the outcomes of scholarly investigations on the learning rate have also been inconsistent. For example, Dombrovski et al. [11] and Kunisato et al. [9] found that the learning rate was not associated with depressive symptoms in reversal-learning tasks. However, Chase et al. [12] also found that the learning rate did not change after reversal; rather, the learning rate during stimulus discrimination before reversal was lower for depressed individuals than for healthy individuals. 

These inconsistencies in the relationship between depression and reinforcement learning parameters may be due to various reasons. However, a better focus may be on the individual differences in cognitive information processing in reinforcement learning related to depression. In the present study, we focus on interoception to better understand the abnormal reinforcement learning process in depression. Interoception refers collectively to the processing of internal bodily stimuli by the nervous system [13]. Studies have found that perception of one’s heartbeat is dulled in depressed patients [14,15,16] and that the activity of the insula, where interoception is represented [17,18], decreases under varied circumstances, such as rest and emotional experience [19]. 

In reinforcement learning, achieving stability by reducing prediction error and the ensuing surprise experience is assumed to promote learning [5]. As such, the goal of learning is to maintain biological homeostasis through physiological or behavioral change, or allostasis [20]. Such learning causes changes in the body’s internal structures (e.g., the immune, endocrine, and autonomic nervous systems) and in the sensations that emanate as a result of those changes [21]. With regard to the state of human adaptation, we can evaluate our condition vis-à-vis the ideal condition on the basis of these cues [22,23]. This view assumes that affective cues associated with the result of the interoceptive response for a given stimulus such as comfort or discomfort and excitation or rest, are pivotal in the reduction of prediction error [24,25]. Given these postulations, reward-based decision-making may be more directly influenced by state variables such as interoception and feeling than by trait variables such as depression symptoms. Therefore, the effects of interoception and feeling accompanied by interoception on reward reinforcement learning in depression should be considered as a possible cause for inconsistency in reinforcement learning parameters. 

In the present study, we examined the relationship between interoception, depressive feeling, and reinforcement learning parameters. We also examined the relationship between depressive symptoms and reinforcement learning parameters. We hypothesized that low interoceptive awareness and depressive feeling, which are particularly closely correlated with depression, would have negative correlations for the exploration rate, similar to those of depressive symptoms. In addition, to further examine the relationship between interoception and reinforcement learning parameters, we tried to manipulate interoception using biofeedback. We hypothesized that an increase in indicators of interoception (awareness and attention) through this manipulation would be associated with an increase in the exploration rate in reinforcement learning.

## 2. Materials and Methods

### 2.1. Participants and Design

**Figure 1:**
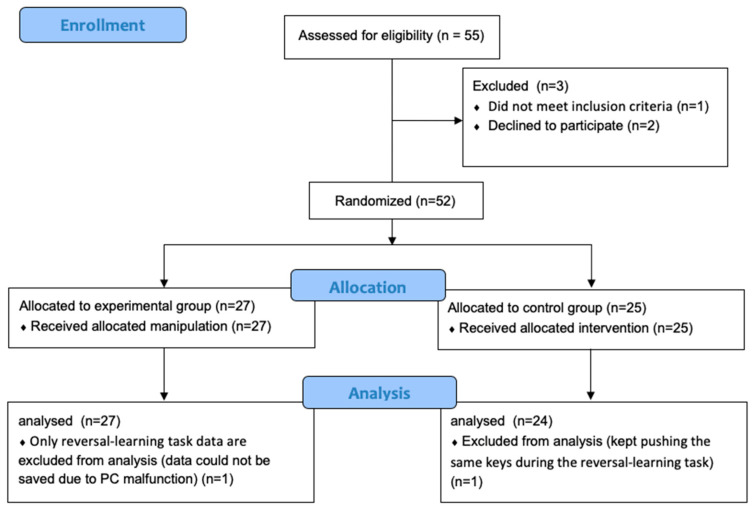
Flow diagram of the progress of participants through the study.

Recruitment advertisements were placed on a Japanese university campus, and 52 undergraduate students were recruited. We recruited as many participants as possible within the limits of the available financial resources. The resulting sample size was larger than or almost equivalent to the samples studied by relevant previously-conducted investigations (e.g., [9,12]). The participants were randomly assigned to the experimental group (19 women and 8 men, mean age 26.1 ± 8.4) and the control group (18 women and 7 men, mean age 22.8 ± 2.2). Age and gender did not significantly differ between two groups (age: *t*(50) = 1.92, *p* = 0.060; gender: χ^2^ = 0.02, *p* = 0.897). The participants were eligible if they did not exhibit any of the following criteria: current illness or physical disease, history of a diagnosed psychiatric disorder, condition of stress immediately prior to the experiment, currently taking medication, or suffering from severe sleep disturbance or fatigue. In addition, the participants were asked to refrain from alcohol and caffeine for the day of the study. One participant was excluded from the control group due to a misunderstanding of the instructions that led him to push the same key many times during the reversal-learning task. The study protocol was approved by the Waseda University ethics committee (2017-008). All participants consented to the study and provided informed consent. Figure 1 shows more details about the progress of participants through the experiment.

### 2.2. Experimental Procedure

**Figure 2:**
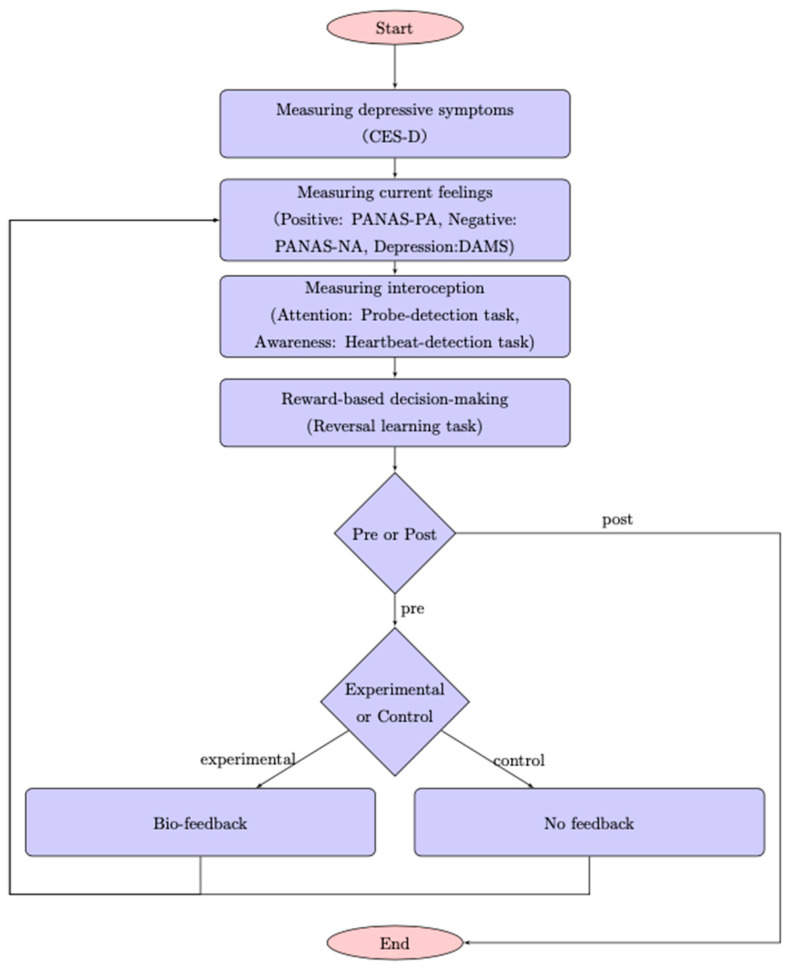
Flow chart of the experimental procedure.

Figure 2 shows the outline of the experimental procedure. At the beginning of the experiment, the Japanese version of the Center for Epidemiologic Studies Depression Scale (CES-D; [26]) was used to assess the levels of depressive symptoms in the participants. The Japanese version of the Positive and Negative Affect Schedule (PANAS; [27]) were used to assess current positive affect (PANAS-PA) and negative affect (PANAS-NA) and the Depression and Anxiety Mood Scale (DAMS; [28]) were also used to current depressive feeling. The DAMS distinguishes between depression and anxiety. It is composed of nine items including items on current depression, anxiety, and positive feeling. In this study, we used only the depression scale (DAMS-D) and this subscale consists of three items, ranging from 3 to 27. The higher the score, the more severe the depressive symptoms. Participants evaluated how true each items was on a 7-point Likert scale, anchored at “strongly disagree” (1) and “strongly agree” (7). Using a parallel test and test–retest method, Fukui [28] found strong convergent validity, discriminant validity, and reliability. In this study, the reliability score for each questionnaire is as follows: pre, α_CESD_ = 0.910, α_PANAS-PA_ = 0.766, α_PANAS-NA_ = 0.931, and D α_DAMS-D_ = 0.910; post, α_PANAS-PA_ = 0.836, α_PANAS-NA_ = 0.885, and D α_DAMS-D_ = 0.780. In our study, electrodes were attached to the participants’ left index fingers and sensors were placed to measure heart rate during the following three experimental tasks: the heartbeat-detection task, the probe-detection task, and the reversal-learning task, performed to measure interoceptive awareness, interoceptive attention, and reward-based decision-making, respectively. Then, the participants in the experimental group were administered a skin-conductance response to help them focus and perceive their inner state more accurately. The control group was instructed to stay relaxed. After the manipulation, the participants completed the PANAS and DAMS assessments and again accomplished all three experimental tasks. All participants were debriefed at the completion of all of the procedures.

### 2.3. Experimental Tasks

#### 2.3.1. Reversal-Learning Task

**Figure 3:**
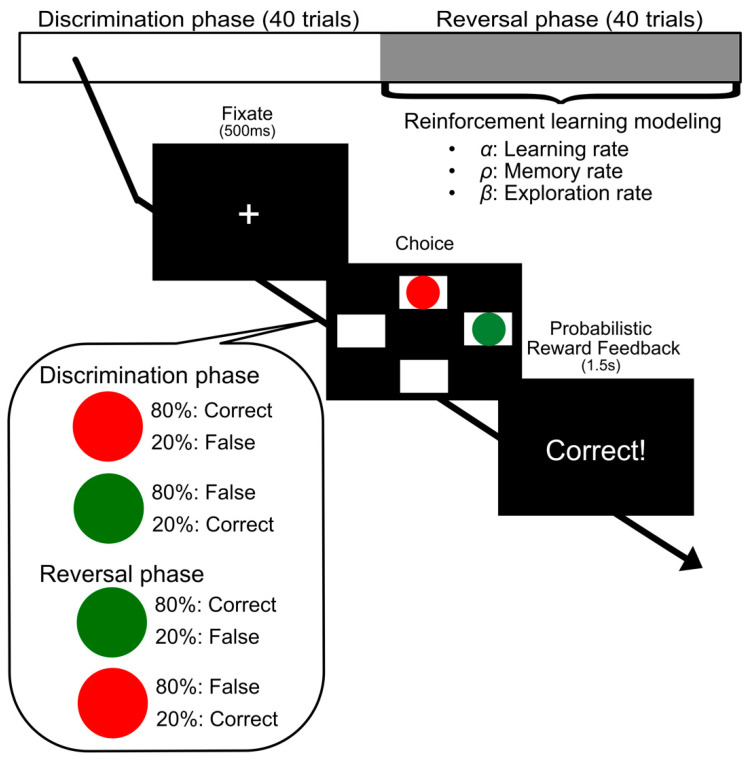
Probabilistic reversal-learning paradigm. The task consisted of 80 trials and involved a single reversal of the stimulus–reward contingencies at the 40th trial. The stimuli varied in color (pre, red and green; post, blue and yellow), and their location (above, below, left, or right) was randomized throughout the task. When participants selected the correct stimulus, they received “correct” or “incorrect” feedback with an 80:20 ratio. When they selected the false stimulus, the ratio was 20:80.

In this task, the two-armed bandit problem was posed to the participants, following Dombrovski et al. [11] (Figure 3). The reversal-learning task has been performed copiously in past research, usually to examine behavior when confronted with the prediction error experience in depression (e.g., [9,11,12,29]). The participants were given instructions for how to perform the task and were informed that the aim of the task was to be as correct as possible. The task consisted of 80 trials and involved a single reversal of the stimulus–reward contingencies at the 40th trial. Thus, 40 trials were included in both the discrimination and reversal phases. However, the participants were not informed of this reversal. Each trial began with the presentation of a fixation cross for 500 ms. Then four quadrants appeared above, below, to the left, and to the right of the central fixation point, and two circular stimuli appeared in two of four quadrants. Participants used the up, down, left, and right keys to select the quadrant with the stimulus which they recognized as correct. The stimuli varied in color (pre, red and green; post, blue and yellow), and their location (above, below, left, or right) was randomized throughout the task. When participants selected the correct stimulus, they received “correct” or “incorrect” feedback with an 80:20 ratio. When they selected the false stimulus, the ratio was 20:80. This indicates that one stimulus had a high reward probability and the other had a low reward probability. The feedback for correct and incorrect choices was given at the end of each trial for a period of 1500 ms. 

#### 2.3.2. Probe-Detection Task

A probe-detection task similar to that of Mansell et al. [30] was used to measure interoceptive attention. This task was developed to measure the balance of online attention between simultaneous internal and external stimuli [30], and it has been used as a paradigm for the examination of internal versus external attention (e.g., [31,32,33]). This task assessed reaction time to an internal probe and an external probe after an emotional stimulus. For the emotional stimuli, we used images of facial expressions, in four categories—anger, happiness, neutrality, and disgust. Each category contained six images (three of men and three of women), with a total of 24 images (abstracted from the standardized set of facial expressions image database developed by Advanced Telecommunications Research Institute International (Database99) [34]). The images were edited to 23 cm by 23 cm and were shown on the screen following a 500 ms presentation of the fixation cross, against a deep green background. The external probe took the form of the letter E written in black, within a yellow green square with a black frame. The internal probe was a vibration that was presented through the electrodes attached to the index finger of the left hand for 100 ms. The vibrations were produced by a linear vibration motor (LD14-002; Nidec Copal Corp., Tokyo, Japan). The probe was presented 2250 ms, 3000 ms, or 3750 ms after the emotional stimulus was shown on the screen, and the participants were asked to press the enter key as quickly as possible when the probe was presented. The task contained four blocks, and one of four facial expression categories was presented in each block. Each block contained 36 trials. The pictures were shown randomly, and the same frequency of sex, image appearance, and probe were ensured in each block. After the outliers were removed using the Smirnov–Grubbs test, interoceptive attention was calculated by subtracting average response time to the internal probe from average responding time to the external probe.

#### 2.3.3. Heartbeat-Counting Task

To assess interoceptive awareness, we adopted Schandry’s heartbeat-counting task [35]. The Schandry’s heartbeat-counting task is the most common method among measures of interoception awareness and has been performed in many studies (a review can be found in [36]). The task had three trials, of 25 s, 35 s, and 45 s. The task began after a 5-s countdown shown on the screen. The participants were instructed to focus on the fixation cross. During the task, the frequency of the actual heartbeat was assessed using an electrocardiogram, while the participants concentrated on their heart rate and reported their count of it. Further, they were prohibited from placing their hands on pulse points or estimating it and restricted to their internal sense alone. The accuracy of the inner sense perception was indexed by calculating the gap between the real heart rate and the subjective heart rate, using the following formula:

(1) $$\sum \frac{\mathrm{actual}\;\mathrm{heart}\_\mathrm{beats}\;-\;\mathrm{reported}\;\mathrm{heart}\_\mathrm{beats}}{\mathrm{Actual}\;\mathrm{heart}\_\mathrm{beats}}$$

### 2.4. Intervention Procedure

#### 2.4.1. Experimental Group

In the experimental group, the participants were given skin-conductance response (SCR) feedback for 10 min. Before beginning the procedure, the participants were informed that SCR is related to the sympathetic nerves. The participants were asked to hyperventilate for few seconds while observing the display to show how the SCR waves on the screen change. Then the participants were directed to report in which body part they could best perceive the sweat glands and muscles and to concentrate on that part to isolate the somatic sensations they were feelings at the moment. However, as in this study we were intending to alter perceptual and attention tendencies, we instructed the participants not to intentionally inhibit or emphasize their reactions on purpose and to simply observe the waveforms to allow them to pay closer attention to their body senses. All body parts that can feel excitement or relaxation could be used as the part the participants were to concentrate on. They were instructed to remain as still as possible during the feedback to minimize motion noise.

#### 2.4.2. Control Group

The participants were fitted with electrodes, were not presented any feedback, and were simply told to remain still and watch the monitor for 10 min.

### 2.5. Electrophysiological Recording

#### 2.5.1. Skin Conductance Response

The participants were fitted with non-polarizable Ag–AgCl adhesive disposable electrodes (Ambu A/S, Copenhagen, Denmark) on the medial phalanx of the right hand index and middle fingers and the wrist of the right hand. SCR was amplified by a SS-WS1107 unit (Sports Sensing Corp., Fukuoka, Japan), which acquired data at 250 Hz and was connected to a PC running AcqKnowledge software (Sports Sensing Corp., Fukuoka, Japan).

#### 2.5.2. Heart Rate

The participants were fitted with non-polarizable Ag–AgCl adhesive disposable electrodes (Nihon Kohden, Tokyo, Japan) attached to the medial phalanx of the right hand index and middle fingers and the wrist of the right hand. The EEG was amplified by a PEG-1000 unit (Nihon Kohden, Tokyo, Japan), acquiring data at 512 Hz, connected to a PC. HR was calculated using the software built into the PEG-1000 unit.

### 2.6. Reinforcement Learning Parameter

#### 2.6.1. Experience-Weighted Attraction Model

The experience-weighted attraction (EWA) model [37] was adopted as the reinforcement learning model. This model was an augmented version of a standard Rescorla–Wagner model of learning and developed to provide a comprehensive assessment of both the reinforcement learning and belief learning models. When specific parameter values are set, an equation that is identical to the Rescorla–Wagner template and the belief learning model, is generated. In comparison to TD learning or Q-learning, the EWA model can estimate the learning rate, while it takes the experience weight into account. For instance, the memory rate, which is a learning parameter reflecting experience weight, is assumed to be crucial in explaining the learning deficit that occurs in depression [3,11]. Because of this feature, the EWA model has been applied to reversal-learning tasks in previous studies [11,38].

In EWA learning, the attractiveness of behavioral choices is continuously updated as choices are weighted based on the rewards produced. In the EWA model, two variables are updated for each trial. The first is *N* (*t*), which determines the ‘experience weight’ of any trial *t* and is represented by the following formula:

(2) N (t)= ρN (t−1)+1

here, *N (0)* = 1, and ρ denotes the depreciation rate according to which previous experience is discounted. In other words, if ρ = 0, previous experience is not taken into account at all and decisions, therefore, are made only by the results of the last trial, while if ρ = 1, all previous experience is taken into account and decisions, therefore, are made by the results of all trials.

The second is $A_{i}^{j}$ *(t)*, which describes the attractiveness of the behavior $s_{i}^{j}$ for which stimulus *j* is selected by any participant *i* in a certain trial *t* and is represented by the following formula:

(3) $$A_{i}^{j}\left(t\right)=\frac{\left(1-\alpha \right)N\;\left(t-1\right)A_{i}^{j}\left(t-1\right)+I\left(s_{i}^{j},\;s_{i}\left(t\right)\right)}{N\;\left(t\right)}$$

here, *A (0)* = 0, and α is Rescorla–Wagner learning rate. α represents the speed at which behavioral values for stimuli are learned. If α = 1, attractiveness is determined entirely from the results of selecting the current stimulus, while if α = 0, past attraction influences current attraction, with no discount. Note that for ρ = 0, *N (t)* is always 1, and the formula is equivalent to the Rescorla–Wagner model. Moreover,$\;I\left(s_{i}^{j},\;s_{i}\left(t\right)\right)\;$ is a function adjusted to increase the attractiveness of the behavior $s_{i}^{j}$ by +1, when $s_{i}\left(t\right)$, the participant *i*’s selection behavior in a certain trial *t*, gets accurate feedback.

#### 2.6.2. Choice Probabilities

In accordance with Camerer and Ho [37], in a certain trial *t*, the choice probabilities, for which option is more likely to be chosen by any participant *i* for stimulus *j* in the next trial *t*, are represented by the following formula:

(4) $$P_{i}^{j}\left(t+1\right)=\frac{{\left(A_{i}^{j}\left(t\right)\right)}^{\beta }}{\sum_{k=1}^{m_{i}}{\left(A_{i}^{k}\left(t\right)\right)}^{\beta }}$$

note that *m_i_* is the number of stimuli and *β* is inverse temperature and describes greediness for attraction. The higher *β* becomes, the greedier about selecting correct stimuli, resulting in lower behavioral randomness. In contrast, the lower *β* becomes, the less greedy about selecting correct stimuli, resulting in higher behavioral randomness.

#### 2.6.3. Parameter Estimation

With the above model formulas, Bayesian hierarchical modeling [7] was used to estimate the parameters. Here, the posterior distributions P(θ|D) of every learning parameter for all participants are described as follows.

(5) $$P(\theta |D)=\frac{P(D|\theta )P\left(\theta \right)}{P\left(D\right)}=\frac{P(D|\theta )P\left(\theta \right)}{\int P(D|\theta )P\left(\theta \right)d\theta_{i}}$$

note that, θ = [α_all_, ρ_all_, β_all_], and D denote all the acquired data. Further, to calculate each parameter of each individual *i*, the hyper-parameters Φ*_i_* are placed as Φ*_i_* = [${µ}_{\theta i}$, $\sigma_{\theta i}$: $\theta_{i}$ = [α*_i_*, ρ*_i_*, β*_i_*], μ is mean, σ is distribution], and the joint posterior distribution *p (Φ_i_ | D_i_)* is described as follows.

(6) $$P\;(\theta_{i},\;\Phi_{i}|\;D_{i})=\frac{P\left(D_{i}|\theta_{i},\;\Phi_{i}\right)P\left(\theta_{i},\;\Phi_{i}\right)}{P\left(D_{i}\right)}\;\propto \;P(D_{i}|\theta_{i})P\left(\theta_{i}|\Phi_{i}\right)P\left(\Phi_{i}\right)$$

note that *p*(*Φ_i_*) represents the prior distribution of the hyper-parameters, $P\left(\theta_{i}|\Phi_{i}\right)$ indicates the prior distribution of the parameters, and $P(D_{i}|\theta_{i})$ reflects the likelihood of the data estimated from the given parameters. In this study, we followed Ann et al. [7] and calculated the prior distribution of parameters as ${µ}_{\theta }$ ~ Normal (0, 10), $\sigma_{\theta }$ ~ half–Cauchy (0, 5) and $\theta_{i}$ ~ Normal ($\mu_{\theta }$, $\sigma_{\theta }$). The likelihood was estimated through sampling, using the NUTS–Hamilton Monte Carlo method, a type of Markov chain Monte Carlo approach (MCMC). After obtaining the posterior distribution of each parameter, we used the maximum of the posteriori estimate (MAP estimate), which is the point that maximizes the value of the posterior distribution as the point estimation. An MCMC sample was performed for four chains (i.e., 4 × 1000 steps), and it was confirmed by using trace plot that the posterior distribution converged to similar values and distributions in all chains.

#### 2.6.4. Fitness of Data

The fitness of data was also confirmed using widely-applicable information criteria (WAIC; [39]). *WAIC_pre_* = 3533.615 in the pre-test and *WAIC_post_* = 3746.354 in the post-test.

#### 2.6.5. Data Modeling

Following Dombrovski et al. [11], we carried out reinforcement learning modeling based on the EWA model for behavioral data during the reversal phase (i.e., the last 40 trials) at the end of the task (80th trial). Similarly, following Dombrovski et al. [11], parameters ρ, α, and β were defined as learning rate (α), memory rate (ρ), and exploration rate (β).

### 2.7. Data Analysis

All statistics were calculated using R (version 3.2.0, The R Foundation for Statistical Computing 2019, https://www.r-project.org/) and a posterior inference for all models was performed with a MCMC sampling scheme using RStan. Group differences were investigated using *t* tests. Pearson’s correlation coefficient was calculated for reinforcement learning parameters and each indicator (depressive symptom, interoception, and feeling). Proportional data (interoceptive awareness) were analyzed as described above after the first arcsine transformation was performed to approximate normal distribution. Values of *p* < 0.05 were considered to indicate statistically-significant differences [40].

## 3. Results

### 3.1. Preliminary Analysis 

Participants’ baseline data are shown in Table 1, and t tests were performed to detect group differences in baseline scores on the subject scales (the Center for Epidemiologic Studies Depression Scale (CES-D), the Positive and Negative Affect Schedule (PANAS), and the Depression and Anxiety Mood Scale (DAMS)) and behavioral data (heartbeat-detection task and probe-detection task), as well as the reinforcement learning parameters (α, ρ, and β). There was no group difference in these scores. Additionally, we investigated the effect of the biofeedback manipulation in the experimental group on interoception (attention and awareness) to explore the hypothesis that manipulation improves interoceptive awareness (Table 1). Significant interactions of group and time on interoception were not found for any indicator (*F*s < 1.024, *p*s > 0.317), which suggested we could not successfully manipulate interoception. Therefore, we performed following analysis without considering the effect of group, and our hypotheses were tested using only the pre-test data. In addition, based on the finding that women show lower interoception accuracy [41,42], we conducted a post-hoc analysis to examine the possibility that gender differences may influence results as individual differences. No effects of gender differences were observed for any of the interoception and feeling parameters (all *p*s > 0.175).

### 3.2. Correlations between Depressive Symptoms and Reinforcement Learning Parameters in the Pre-Test Results

We investigated the correlation between CES-D scores and the reinforcement learning parameters α, ρ, and β to explore the hypothesis that depression symptoms correlate with the exploration rate in particular. CES-D scores negatively correlated with β, although this did not reach significance (*r =* −0.248, *p* = 0.098; Table 2).

### 3.3. Correlations between Interoception/Feelings and Reinforcement Learning Parameters in the Pre-Test Results

We investigated the correlation between interoception (attention and awareness) and the reinforcement learning parameters α, ρ, and β to explore the hypothesis that interoceptive awareness in particular is related to the exploration rate (Table 2). A significant, weakly to moderately positive correlation was found with β for interoceptive attention (*r* = 0.368, *p* = 0.010). Interoceptive awareness was also weakly and positively associated with β, while it did not reach significance (*r* = 0.234, *p* = 0.099). On the other hand, the positive correlation between awareness and β showed only a weakly-significant trend.

Next, we investigated the correlation between feeling indicators (the scores for the DAMS-D, PANAS-PA, and PANAS-NA) and the reinforcement learning parameters α, ρ, and β to examine the hypothesis that depressive feelings in particular is related to the exploration rate (Table 2). A significant, weakly to moderately negative correlation was found with β for DAMS-D score (*r* = − 0.419, *p* = 0.002). The scores for the PANAS-PA demonstrated a significant, weakly to moderately positive correlation with α (*r* = 0.368, *p* = 0.008).

## 4. Discussion

In this study, we investigated reward-based decision-making, using reinforcement learning modeling, and explored the role of interoception and depressive feelings in this context. The results indicated that higher levels of depressive symptoms were associated with lower exploration rate β, although the significance was marginal. Additionally, interoceptive attention and depressive feelings had a significant correlation with exploration rate β. These findings partially support our hypothesis that interoception and depressive feelings are positively correlated with exploration rate β.

Our finding suggested the possibility that it is not the depressive symptom itself but the status of one’s feelings that forms the basis of reward-based decision-making impairment. It is known that the presence of a negative mood generates a negative bias of attention, memorizing, learning, and paying attention to only negative stimuli selectively [43,44,45]. Also, stimuli consistent with one’s current feelings are more likely to be consistent with predictions and making them easier to learn [21]. As such, individuals with depressive feelings are more susceptible to the influence of punishing stimuli and the randomness of their select behavior is exacerbated (i.e., β decreases). The validity of this interpretation is corroborated by the result of this study that positive feelings were correlated with learning rate α. Further, as per previous findings, when individuals with severe depression are presented with both neutral and negative stimuli, they selectively remember only the negative stimuli and have difficulty suppressing the encoding of such stimuli [46].

At the same time, the relationship between depressive feeling and decision-making instability may be related to interoception processing. In fact, the lower an individual’s interoceptive attention, the higher the randomness of their behavior (i.e., lower β). Interoception is one of the cues for prediction error detection and affects the emotional decision-making process. Thus, it is especially important for the tasks with rewards. For example, Bechara et al. [47] found that patients with the damage to the ventral medial frontal cortex, which is known to generate physical responses properly during decision-making, show problems in predicting long-term rewards. They tended to choose the gamble stimulus for short-term gains despite the possibility of long-term losses. The present study supports this finding, and if individuals with high levels of depression fail to properly code reward-prediction error by focusing attention to one’s internal state, they are more likely to be influenced by punishment feedback because they may have difficulties in encoding long-term gains. It is also important to note that contrary to conventional theory, a significant relationship between interoceptive awareness and exploration rate β was not observed. This is possibly because intuitive decision-making tasks, as in the present study, do not require a subjective understanding of one’s internal states, such as awareness. However, it is also known that awareness as an information-processing process builds on attention [48]. Therefore, when considering the complex decisions that we make on a daily basis, the impact of interoceptive processing should also be considered.

In summary, it is suggested that state variables such as interoception and feeling may be more closely involved in the decision-making of reward exploration and value learning. Thus, although previous studies have not found consistent results for the correlation between depression and reinforcement learning parameters, this may be because the reinforcement learning parameters were in turn reflecting feelings, which change easily. However, the above understanding is, ultimately, only a suggestion. We also carried out biofeedback manipulation in the experimental group to investigate whether manipulation of interoceptive awareness, interoceptive attention, or feelings had an effect on reward-based decision-making. However, our biofeedback procedure was not successful in that no significant changes were observed in interoceptive awareness, interoceptive attention, and feelings after manipulation.

There are at least three possible reasons why biofeedback manipulation did not have an effect on interoception or feeling. The first relates to the biofeedback method. We instructed the participants to simply observe the state that their body was currently in without intentionally trying to manipulate the SCR waveform. This is because the goal was not to reach a specific physical state but to focus attention and awareness on the physical state brought of the body. This method differs from the conventional biofeedback method, in which participants are instructed to bring the waveform within a certain range [49]. Nonetheless, because interoceptive awareness did not increase as hypothesized, it may be that the manipulation used in the current study was inadequate. The second potential reason is from the influence of practice effects. In this study, participants repeated the same task within a relatively short period of time, and it is possible that the effects of growing accustomed to and practicing the task strongly influenced the results of the post-intervention measurement. We adopted the above paradigm to investigate the effects of manipulating the situational variables for interoception and feeling, but adjustments such as revising the schedule of biofeedback intervention and post-intervention measurements may have been necessary. The third reason is the meditation level of the participants. In this study, we did not measure the meditation level and did not exclude participants who experienced meditation, such as mindfulness and yoga. However, given the relatively large variance in the interoceptive variables at baseline in this study, it may be necessary to consider the influence of variables such as the participants’ meditation level, which is assumed to be one of these factors. In fact, previous studies have shown that those who have practiced meditation have high attention and awareness level (e.g., [50]) and, consequently, there is a possibility that their mixing made the operation ineffective in this study. Furthermore, mindfulness meditators show an increase in the posterior insula activity when they experience prediction error, which means interoceptive awareness has been promoted [51]. Hence, in the future, it will be necessary to control for meditation levels or to compare between different meditation levels to test the effectiveness of interoceptive manipulation.

### Limitations

First, although this study examined the effect of interoception and feelings in decision-making, these interoceptive and feeling indicators were measured before the intervention and not during the reversal-learning task. Accordingly, we were unable to consider whether the given values of these variables truly influenced reinforcement learning parameters during task performance. Interoception in particular is thought to be involved in the detection of surprise experience (i.e., prediction error) as a cue to one’s status, including comfort or discomfort and rest or arousal [21]. Therefore, it may be essential to measure participant status specifically during their decision-making. Nonetheless, more recently, indicators that can be measured simultaneously with other tasks have been proposed, such as heartbeat-evoked potentials as indicators of interoception [52]. In future research, it will be necessary to adopt this type of measurement to investigate the potential effects of interoception and feelings in decision-making.

We should also be cautious about the reliability of the tasks used in this study to measure these indicators. These tasks have been widely used in previous studies, however, their reliability was not sufficiently considered. For example, it must be pointed out that the heartbeat-counting task used to evaluate interoceptive awareness registered different results for the same participant in Zamariola et al. [53], depending on the duration of the trial time, and the score tended to decrease as the heart rate increased. Prospective research projects need new measurement tools to more reliably assess the constructs measured by this study.

Third, we assumed that decision-making in the reversal-learning task in this study would be controlled by an accompanying reward. However, it has been suggested that the high behavioral randomness (low exploration rate β) found in depression in this study may have resulted from an avoidance of probabilistically-presented punishments. To avoid this issue, future research must consider the role of punishment in reward-based decision-making, such as by adopting a method that calculates the learning rate for both rewarding stimuli and punishing stimuli [38].

Lastly, we did not conduct a-priori power analysis to determine an adequate sample size. In the future, it is desirable to conduct experiments based on the effect of biofeedback that has been clarified in this study.

## 5. Conclusions

In this study, we were unable to explore causal effects of interoception and feelings on the decision-making process. Meanwhile, the findings in the pre-test suggest that abnormal reward-based decision-making can be understood in relation to the general decision-making principle of prediction error by considering interoception and feelings. Therefore, future research may be worthwhile to address this causal issue through interventions for decision-making difficulties in depressive symptoms.

In fact, past studies has shown that mindfulness, which enhances emotional regulation by increasing the ability to control one’s attention to physical resources [50], improves depression, negative feeling, and heuristic bias [54] as well as enhancing intellectual decision-making by collecting bottom-up interoception information [55]. In depression, it is found that there is a rather low capacity for self-understanding based on physical status [21]. Therefore, in depression, there is a possibility that improving the understanding of the interoception and the accompanying feelings through mindfulness may be effective in improving reward decision-making. Thus, it will be necessary for future work to improve methods of increasing interoceptive awareness and stabilizing feelings and to evaluate the ways in which interoception and feelings influence decision-making with longer-term interventions.

## Figures and Tables

**Table 1:** Means (standard deviations) of variables at pre- and post-test.

	Pre			Post	
	Experiment	Control	*t* Value	Experiment	Control
Depressive Symptom					
CES-D	17.74 (11.05)	15.08 (7.23)	*t*(50) = 1.02 (*p* = 0.31)		
Feeling state					
DAMS	8.67 (3.92)	8.70 (3.37)	*t*(50) = 0.38 (*p* = 0.71)	6.44 (2.87)	6.32 (3.36)
PANAS-PA	29.81 (5.25)	29.44 (7.05)	*t*(50) = 0.22 (*p* = 0.83)	26.78 (6.88)	23.28 (6.74)
PANAS-NA	23.15 (10.03)	22.08 (7.69)	*t*(50) = 0.43 (*p* = 0.67)	18.26 (7.57)	16.84 (7.13)
Intereception					
Awareness	0.56 (0.29)	0.61 (0.28)	*t*(50) = −0.55 (*p* = 0.58)	0.60 (0.24)	0.67 (0.24)
Attention	0.02 (0.06)	0.03 (0.06)	*t*(50) = −0.68 (*p* = 0.50)	0.03 (0.05)	0.03 (0.05)
Reversal learning					
Learning rate (α)	0.61 (0.19)	0.65 (0.19)	*t*(49) = 0.73 (*p* = 0.46)	0.62 (0.16)	0.65 (0.20)
Memory rate (ρ)	0.31 (0.19)	0.35 (0.24)	*t*(49) = −0.50 (*p* = 0.61)	0.26 (0.13)	0.30 (0.23)
Exploration rate (β)	1.47 (0.46)	1.48 (0.36)	*t*(49) = −0.02 (*p* = 0.99)	1.62 (0.35)	1.62 (0.35)
The number of correct answers	31.11 (4.36)	29.72 (6.70)	*t*(50) = 0.89 (*p* = 0.38)	32.69 (3.41)	31.60 (3.87)
Error rate (before reversal)	0.21 (0.06)	0.24 (0.08)	*t*(50) = −1.02 (*p* = 0.31)	0.20 (0.08)	0.22 (0.09)
Error rate (after reversal)	0.26 (0.10)	0.29 (0.16)	*t*(50) = 0.89 (*p* = 0.38)	0.22 (0.08)	0.25 (0.09)

Note: CES-D, the Center for Epidemiologic Studies Depression Scale; DAMS-D, Depression and Anxiety Mood Scale-depression mood; PANAS-PA, the Positive and Negative Affect Schedule-positive affect; PANAS-NA, The Positive and Negative Affect Schedule-negative affect. Note: Interoceptive awareness was measured by a heartbeat-detection task and interoceptive attention was measured by a probe-detection task.

**Table 2:** Correlations between reinforcement learning parameters and variables in the pre-test.

	Symptom	Mood			Interoception	
	CES-D	DAMS-D	PANAS-PA	PANAS-NA	Attention	Awarness
Symptom						
CES-D		0.702 *** (*p* = 0.000)	−0.244 ^†^ (*p* = 0.081)	0.547 *** (*p* = 0.000)	−0.053 (*p* = 0.714)	−0.093 (*p* = 0.513)
Reversal learning parameters						
Learning rate (α)	−0.093 (*p* = 0.514)	−0.099 (*p* = 0.488)	0.368 ** (*p* = 0.008)	0.176 (*p* = 0.217)	0.178 (*p* = 0.216)	0.005 (*p* = 0.970)
Memory rate (ρ)	−0.083 (*p* = 0.561)	0.124 (*p* = 0.386)	−0.054 (*p* = 0.707)	−0.033 (*p* = 0.818)	−0.116 (*p* = 0.423)	−0.021 (*p* = 0.885)
Exploration rate (β)	−0.240 ^†^ (*p* = 0.090)	−0.419 ** (*p* = 0.002)	0.234 ^†^ (*p* = 0.099)	−0.038 (*p* = 0.791)	0.361 * (*p* = 0.010)	0.233 ^†^ (*p* = 0.099)

*** *p* < 0.001, ** *p* < 0.01, * *p* < 0.05, ^†^ *p* < 0.10 Note: CES-D, the Center for Epidemiologic Studies Depression Scale; DAMS-D, Depression and Anxiety Mood Scale-depression mood, PANAS-PA, the Positive and Negative Affect Schedule-positive affect, PANAS-NA, the Positive and Negative Affect Schedule-negative affect. Note: Interoceptive awareness was measured by heartbeat-detection task, and interoceptive attention was measured by the probe-detection task.
